# Theoretical and Experimental Studies of the Structural, Phase Stability and Elastic Properties of AlCrTiFeNi Multi-Principle Element Alloy

**DOI:** 10.3390/ma13194353

**Published:** 2020-09-30

**Authors:** Li Liu, Ramesh Paudel, Yong Liu, Xiao-Liang Zhao, Jing-Chuan Zhu

**Affiliations:** 1 School of Materials Science and Engineering, Harbin Institute of Technology, Harbin 150001, China; lyonghit@hit.edu.cn (Y.L.); hitzhxl@163.com (X.-L.Z.); 2 Nepal Academy of Science and Technology (NAST), Khumaltar, Lalitpur 44700, Nepal; r.paudel@hit.edu.cn; 3 National Key Laboratory of Science and Technology on Advanced Composites in Special Environments, Harbin Institute of Technology, Harbin 150080, China

**Keywords:** AlCrTiFeNi multi-principle element alloy, structure, micro mechanism

## Abstract

The fundamental challenge for creating the crystal structure model used in a multi-principle element design is the ideal combination of atom components, structural stability, and deformation behavior. However, most of the multi-principle element alloys contain expensive metallic and rare earth elements, which could limit their applicability. Here, a novel design of low-cost AlCrTiFeNi multi-principle element alloy is presented to study the relationship of structure, deformation behavior, and micro-mechanism. This structured prediction of single-phase AlCrTiFeNi by the atomic-size difference, mixing enthalpy $\Delta H_{mix}$ and valence electron concentration (VEC), indicate that we can choose the bcc-structured solid solution to design the AlCrTiFeNi multi-principle element alloy. Structural stability prediction by density functional theory calculations (DFT) of single phases has verified that the most advantageous atom occupancy position is (FeCrNi)(AlFeTi). The experimental results showed that the structure of AlCrTiFeNi multi-principle element alloy is bcc1 + bcc2 + L_12_ phases, which we propose as the fundamental reason for the high strength. Our findings provide a new route by which to design and obtain multi-principle element alloys with targeted properties based on the theoretical predictions, first-principles calculations, and experimental verification.

## 1. Introduction

Multi-principal high-entropy alloys are composed of five or more elements in an equimolar ratio, and the atomic percentage of each component does not exceed 35% [1,2]. Due to their unique performance advantages, such as high strength [3], high hardness [4], high wear resistance [5], and good resistance to high temperature softening [6], multi-principal alloys are an alloy type with great academic research value and industrial application potential in the field of materials science [7]. The application of multi-principal alloys involves many areas, such as tools, abrasives, and mechanical parts. Since the concept of multi-principal element alloy proposed, many experts and scholars have researched the related fields of multi-principal element alloys, such as their microstructure, composition phase, and mechanical properties. Some multi-principal alloys have complex microstructures, such as the composition phase, including many intermetallic compounds, making them brittle and difficult to handle and analyze. Moreover, some multi-principal alloys are composed of a simple body-centered cubic structure bcc, a face-centered cubic structures fcc, a close-packed hexagonal structure, or a dual-phase structure consisting of several systems [8,9,10]. They have excellent performance characteristics, such as high strength and ductility. Therefore, the formation of multi-principal alloys is an interesting scientific problem.

Although there are many kinds of elements that make up a multi-principle alloy, their phase space is still limited. It has been reported that most of the multi-principal element alloys use a single-phase structure of metal elements as the constituent elements, and have a relatively uniform atomic size and similar electronic structure [11,12]. Most of the multi-principal element alloys contain expensive elements (cobalt, Nb) or rare earth elements (Sc, Y, and lanthanides). For example, in the past decade, many multi-principal alloys have been reported. For example, L12/B2 dual-phase AlCrCoFeNi alloy with high strength and ultra-high ductility at high temperature [13,14,15]. Also, studies have reported that the strength of CoCrFeNiNb_0.25_ multi-principal alloys is enhanced by the FCC phase [16], and studies have shown that the trace element Nb will affect the as-cast structure and heat-treated structure and properties of CoCrFeNb_x_Ni multi-principal alloys [17,18]. Since most of the multi-principal component alloys contain expensive elements such as cobalt and niobium, their applications are limited. Therefore, the use of low-cost and effective elements instead of expensive elements to develop multi-principal alloys will reduce the cost of alloys and promote their applications.

According to the composition characteristics of the multi-principal element alloy, it is possible to design a new type of multi-principal element alloy by breaking the traditional cooking-style alloy design concept. Material genetic engineering studies the relationship between the primary constituent elements of materials, structure, organization, and characteristics. By adjusting the way the constituent atoms occupy positions, targeted new materials are obtained. Material-based computational design and simulation have become an important part of current materials science research. Here we have performed a theory to predicts the crystal structure of single-phase AlCrTiFeNi, making an extensive first-principles study to evaluate atom occupancy positions, structural stability, and elastic properties for bcc AlCrTiFeNi multi-principle element alloy, our set of alloys include, in particular, the (CrFeNi)(AlTi), (CrTiNi)(AlFe), (TiCrFe)(AlNi), (TiCrFe)(AlCr), and (FeCrNi)(AlFeTi) atom occupancy positions. Experimentally, we verified the formation of the most stable single-phase bcc structure, and confirmed the structure and microscopic mechanism. Studies have shown that the structure and microscopic mechanism are related to the positions occupied by atoms.

## 2. Materials and Methodology

### 2.1. Theoretical Methods

The atomic size difference δ, the mixing enthalpy of a solid solution $\Delta H_{mix}\;\left(\mathrm{kJ}/\mathrm{mol}\right)$ and valence electron concentrations VEC were introduced in this work to investigate the solid solution characteristics of single-phase AlCrTiFeNi multi-principle element alloys. Here, the parameter δ is used to describe the influence of the atomic size difference in the multi-principal element alloy. The calculation formula is as follows [19]:

$$\delta =\sqrt{\sum_{i=1}^{N}C_{i}}{\left(1-r_{i}\sqrt{r}\right)}^{2}$$

where *N* is the number of element types in the alloy system, $C_{i}$ is the atomic percentage content of the ith element, $r_{i}$ is the average atomic radius, and the average atomic radius *r_i_* can be obtained in the reference [20]

$$\Delta H_{mix}=\sum_{i=1,i\neq j}^{n}\Omega_{ij}C_{i}C_{j}$$

where $\Omega_{ij}\left(=4\Delta H_{AB}^{mix}\right)$ is the conventional melt interaction parameter between the ith element and the jth element, and $\Delta H_{AB}^{mix}$ is the mixing enthalpy of the binary liquid alloy, and its value can be obtained in the reference [21].

### 2.2. Calculation Methods

First-principles calculations are performed by a plane-wave pseudo-potential approach selected the PBE framework in the generalized gradient approximation (GGA) [22,23], as implemented in the CASTEP code. Structures were fully relaxed in the lattice parameters and internal coordinates at T = 0 K using the energy cutoff of 350 eV and the k-point meshes were determined according to the Monkhorst–Pack scheme. Structures were fully relaxed in lattice parameters and the single-phase AlCrTiFeNi was built based on a simple supercell with BCC unit cells [22]. The atom occupancy positions due to the Miedema theory [24,25], where the atoms in their positions are fully distributed, and the nearest-neighbor atoms must be inconsistent. Both the volumes and atomic positions of the supercells were optimized until the average force on each atom, displacement, stress, and energy change were below 0.01 eV/Å, 5.0 × 10^−4^ Å, 0.02 GPa, and 5.0 × 10^−6^ eV/atom, respectively. Predict single-phase elastic constants can be expanded to predict structural stability based on Born stability criteria [26]:

$$C_{11}>0,\;C_{22}>0,\;C_{33}>0,\;C_{44}>0,\;C_{55}>0,\;C_{66}>0,(C_{11}+C_{33}-2C_{13})>0,\;(C_{22}+C_{33}-2C_{23})>0,$$

$$\left(C_{11}+C_{22}-2C_{12}\right)>0,(C_{11}+C_{22}+C_{33}+C_{12}+C_{13}+C_{23})>0,$$

These polycrystalline structural properties can be estimated in terms of single-crystal elastic constants:

(1) $$B=\frac{B_{V}+B_{R}}{2},\;G=\frac{G_{V}+G_{R}}{2},\;E=\frac{9BG}{3B+G},\;\nu =\frac{3B-E}{6B}$$

### 2.3. Experimental Method

The material selected in this paper is AlCrTiFeNi alloy which is made by vacuum arc smelting and consists of elements (Al, Cr, Ti, Fe, Ni, purity: 99.9 wt.%). Selected the alloy of the same composition (each element content is 20%) to smelt 50 g/piece of button material. The equipment used is FD-1200 H non-consumable high-vacuum arc melting furnace, which adopts water-cooled copper crucible to smelt alloy by ionizing argon ion to start arc.

**Figure 1:**
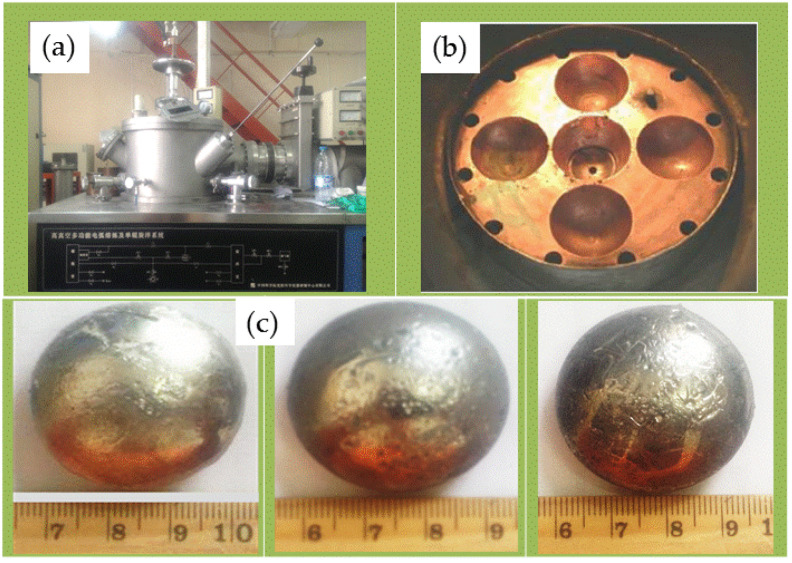
Schematic diagram of material preparation process: (**a**) High vacuum multifunctional arc melting and single-roll spin quenching system; (**b**) Water-cooled copper crucible-smelting tank; (**c**) Button material after smelting.

During alloy smelting, in order to ensure that the elements of different melting points are completely melted, and the alloy is smelted uniformly, the order of placing the raw materials in the water-cooled copper crucible is from the lowest melting point to the highest. The lowest layer is the element with the lowest melting point, and the uppermost layer is the highest melting point. In the smelting process, in order to prevent the alloy from being oxidized, the non-consumable vacuum arc furnace body is evacuated to a vacuum degree below 5.0 × 10^−3^ MPa, and then filled with high-purity argon to make the furnace body vacuum degree to 0 MPa. Start arc smelting at, the current is slowly increased from 0 A to about 350 A, and the final smelting current is maintained between 250–300 A. After smelting for about 2 min, the alloy ingot is turned over and smelted again. Each alloy sample is repeatedly smelted 4 times. To ensure uniform melting of the alloy. Figure 1 is a schematic diagram of the material preparation process.

Three samples of 10 × 10 × 10 were selected, and the alloy was identified by X-ray diffraction and Cu target. The as-cast microstructures of the alloys were characterized by a scanning electron microscopy (SEM) SUPRA 55 SAPPHIRE (Carl Zeiss AG, Oberkochen, Germany). The chemical compositions and microstructure of phases in the alloys were analyzed by SEM-energy dispersive spectrometry (EDS) (Carl Zeiss AG, Oberkochen, Germany) and transmission electron microscopy (TEM)-EDS (FEI Corp., Hillsboro, OR, USA). To analyze the dynamic recrystallization behavior of the alloy after hot deformation processing, the pieces were cut from the recrystallized samples perpendicular to the rolling direction, and then metallographically polished in stages to a final surface. For scanning electron microscope analysis, operated at 30 kV in the back-scattered electron mode, the surface of the sample was electrolytic polishing using a standard solution (10 mL HClO_4_, 90 mL CH_3_COOH).

## 3. Results and Discussion

### 3.1. Structural Stability Prediction

Many kinds of microstructure prediction of multi-principle element alloys have been studied in terms of their atomic-size difference δ [27,28], mixing enthalpy $\Delta H_{mix}$ and valence electron concentration VEC [29,30] since the concept was first proposed. In this work, the solid solution structure prediction of single-phase AlCrFeNiTi multi-principle element alloy was investigated. According to the statistical results [31], when δ < 9.0 is satisfied, a solid solution with bcc structure will be stable. Fitting an equation [32] to the refined atomic-size difference of δ = 7.212, which is related to the formation of the bcc structure solid solution, is in agreement with the literature [33]. The selection of the bcc-phase solid solution can be dependent on not only atomic-size difference results but also a reasonable phase formation criterion. The other parameter, i.e., mixing enthalpy ($\Delta H_{mix}=-0.24$ KJ/mol for this alloy), is generally regarded as the most effective parameter (when −5 KJ/mol < $\Delta H_{mix}$ < 5 KJ/mol) to form the stable solid solution (distinguish compounds) [34]. Previous works show that as-cast multi-principle element alloys will form stable single-bcc, duplex fcc/bcc, and single-fcc structure when VEC < 6.87, 6.87 ≤ VEC < 8.0, and VEC ≥ 8.0 [35]. This is consistent with the single-phase structure predicting in VEC = 6.20(belongs to bcc structure) in this study and previous research of multi-principle element alloys. By summarizing the atomic-size difference δ, mixing enthalpy and VEC criterion simultaneously, we can easily and effectively choose a bcc-structured solid solution for designing AlCrTiFeNi multi-principle element alloys.

**Figure 2:**
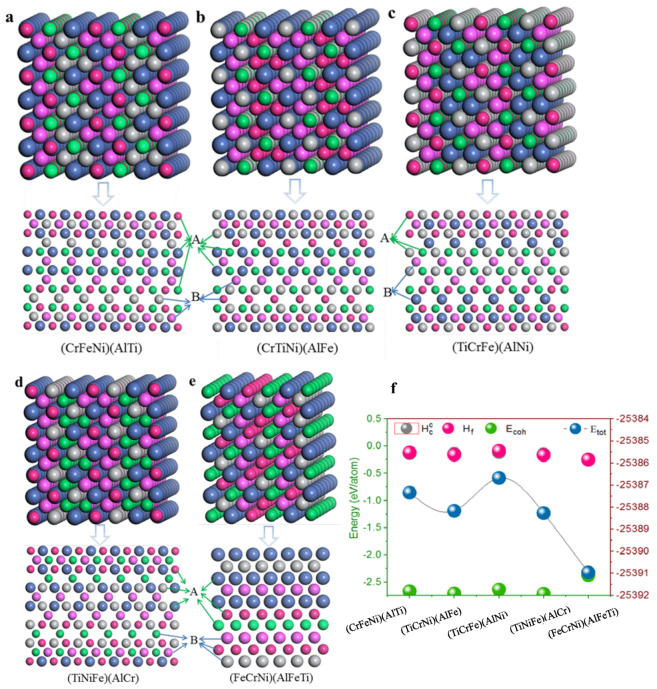
Schematic representation of crystal cell, (110) plane for single-phase AlCrTiFeNi of different atom occupancy positions (‘A’ and ‘B’ represent the atoms position in parentheses: (**a**) (CrFeNi)(AlTi); (**b**) (CrTiNi)(AlFe); (**c**) (TiCrFe)(AlNi); (**d**) (TiCrFe)(AlCr); (**e**) (FeCrNi)(AlFeTi); (**f**) The calculated total energy $E_{\mathrm{tot}}$, formation enthalpy $H_{f}$, cohesive energy $E_{c}$, and secondary solid solution formation enthalpy $H_{c}^{c}$.

Using structural stability prediction by density functional theory calculations of single-phase (CrFeNi)(AlTi), (TiCrNi)(AlFe), (TiCrFe)(AlNi), (TiCrFe)(AlCr), and (FeCrNi)(AlFeTi) different atom occupancy positions, the crystal structures of single-phase AlCrFeNiTi alloys (Figure 2) were built based on a bcc supercell with orthorhombic structure [36]. The atom position A, as well as position B, is equivalent to the crystal structures of single-phase AlCrTiFeNi, and different atoms were placed in the given sites to build the corresponding single-phase AlCrFeNiTi alloys. The lattice constant is a basic physical parameter that may be used to assess the reliability of the crystal structure model, the optimized structural parameters can be simplified as an arithmetic average of lattice constants $a_{avg}$($a_{avg}=\frac{1}{n}\sum_{i=1}^{n}=\frac{1}{3}\left(a_{i}+b_{i}+c_{i}\right)$, where $a_{i},b_{i}\;\mathrm{and}\;c_{i}$ lattice constants of ith unit cells in created supercell are after structure optimization, respectively. n is the number of unit cells in the supercell. The calculated lattice constant by Isostatic deformation method for (CrFeNi)(AlTi), (TiCrNi)(AlFe), (TiCrFe)(AlNi), (TiCrFe)(AlCr) and (FeCrNi)(AlFeTi) atom occupancy positions are all of a = 2.866 Å, these values are similar to those predicted by previous calculations (a = 2.92 Å).

According to the equation of the formation enthalpy $H_{f}$ and the cohesive energy $E_{c}$ [37], the lower the formation enthalpy and the bigger the cohesive energy, the more stable the crystal structure. The lowest formation enthalpy $H_{f}$ and the larger the cohesive energy $E_{c}$ of (FeCrNi)(AlFeTi) atom occupancy positions, the greater the energy of the other atom occupancy positions. This may be the cause of structural stability. In contrast to the formation enthalpy and the cohesive energy, similar conclusions by Miedema [38] predict a secondary solid solution formation enthalpy $H_{c}^{c}$ of single-phase AlCrTiFeNi. However, they predict that this structure will have a strong sensitivity to energy, indicating that the (FeCrNi)(AlFeTi) atom occupancy positions stabilize the bcc phase of AlCrTiFeNi. This behavior suggests that the atom occupancy positions of this multi-principle element alloy might enhance the stability of the bcc phase for its formation, facilitating phase manipulation.

### 3.2. Elastic Properties and Deformation Behavior Prediction

**Figure 3:**
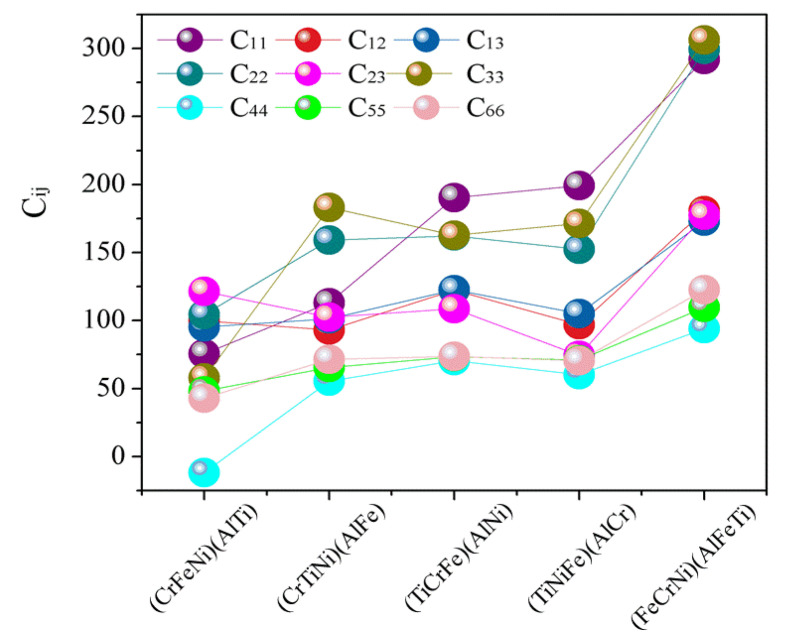
The calculated elastic constants (Cij) for single-phase AlCrTiFeNi of different atom occupancy positions.

The elastic constants predicted by the first-principles calculations and Born stability criteria [39] are directly applied in the present structural stability analysis. The calculated elastic constants for AlCrTiFeNi are listed in Figure 3. If the values of ($C_{11}$) and ($C_{22}$) are very large, suggesting that it is difficult to compress the AlCrTiFeNi alloy under the uniaxial stress along the X ($e_{11}$) or Z ($e_{22}$) axis. As shown in Figure 3, it can easily be seen that, with the exception of the (CrFeNi)(AlTi) atom occupancy positions, the calculated (*C_ij_*) for single-phase (TiCrNi)(AlFe), (TiCrFe)(AlNi), (TiCrFe)(AlCr), and (FeCrNi)(AlFeTi) atom occupancy positions satisfy the Born stability criteria, suggesting that the crystal structure of (CrFeNi)(AlTi) atom occupancy positions are mechanically unstable and hard to be formed, while the (TiCrNi)(AlFe), (TiCrFe)(AlNi), (TiCrFe)(AlCr), and (FeCrNi)(AlFeTi) atom occupancy positions are mechanically stable.

**Figure 4:**
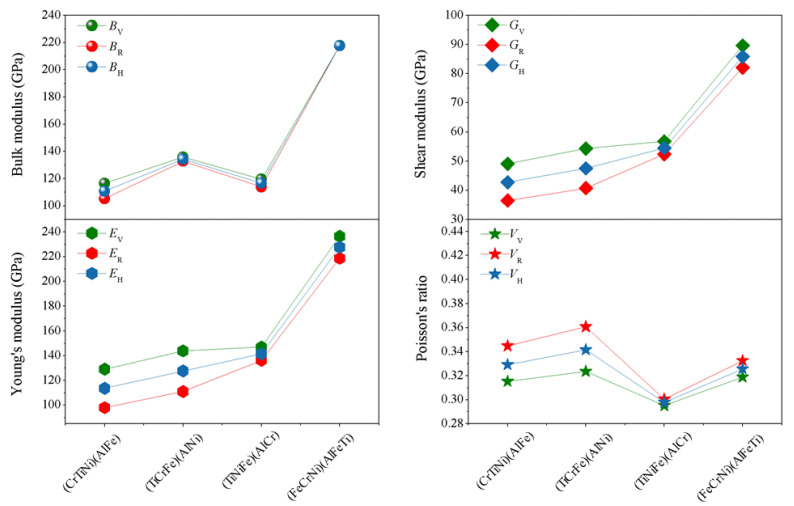
The calculated average properties (bulk modulus (*B*), Shear modulus (*G*), elastic modulus (*E*) and poisson’s ratio (*ν*)) for single-phase AlCrTiFeNi of different atom occupancy positions by using Voigt-Reuss-Hill approximation. (TiCrNi)(AlFe); (TiCrFe)(AlNi); (TiCrFe)(AlCr); (FeCrNi)(AlFeTi) is the positions occupied by atoms.

Based on the elastic constants (*Cij*), using Voigt-Reuss-Hill (V)-(R)-(H) approximations [40,41], the physical parameters of the different atom occupancy positions for AlCrTiFeNi single-phase such as bulk modulus (*B*), shear modulus (*G*), elastic modulus (*E*) and Poisson’s ratio (υ) can be obtained, as shown in Figure 3 The bulk modulus (*B*) is a measure of resistance to volume change by applied pressure, and shear modulus (*G*) is a measure of resistance to reversible deformations upon shear stress [42]. From Figure 4, for the different atomic occupancy positions, the results obtained by using Voigt–Reuss–Hill approximations to obtain the (*B*), (*G*), (*E*), and (υ) results are less different. The (FeCrNi)(AlFeTi) atom occupancy positions, specially, display a larger bulk modulus (*B*), (*G*), and (*E*), than others, which indicates a resistance to volume change and reversible deformations of the (FeCrNi)(AlFeTi) atom occupancy positions, is enhanced by applied pressure.

Poisson’s ratio (υ), ranging from −1 to 0.5, is usually used to evaluate the stability of the crystal structure against shear force. The greater the Poisson’s ratio, the better the plasticity. Compared with the Poisson’s ratio for the (TiCrNi)(AlFe), (TiCrFe)(AlNi), and (TiCrFe)(AlCr) atom occupancy positions, the obtained value of Poisson’s ratio *V*_V_-*V*_R_-*V*_H_ for (FeCrNi)(AlFeTi) atom occupancy position is larger than (TiNiFe)(AlCr), and it has been shown that the plasticity of (FeCrNi)(AlFeTi) atom occupancy position is better (Figure 4). Furthermore, the ratio of bulk to shear modulus (*B*/*G*), i.e., Pugh’s ratio, can be used as a measure to determine the brittle and ductile nature of the material. The critical value separating ductility from brittleness is about 1.75. If Pugh’s ratio (*B*/*G*) comes out to be greater than 1.75, then the material will be ductile. In this paper, the (FeCrNi)(AlFeTi) atom occupancy position has the largest Pugh’s ratio (*B*/*G* = 2.53), respectively, indicating that the (FeCrNi)(AlFeTi) atom occupancy position is the best ductile. The inverse of Pugh’s ratio is called Frantsevich’s ratio (*G*/*B*). The calculated value of (*G*/*B* = 0.205) for the (FeCrNi)(AlFeTi) atom occupancy position is the smallest which also indicated ductility for this atom occupancy of alloy [43]. These results are in accordance with the conclusions of Poisson’s ratio discussed above. The variation of the atom occupancy position changes the mechanical properties of this alloy.

### 3.3. Elastic Anisotropy

**Figure 5:**
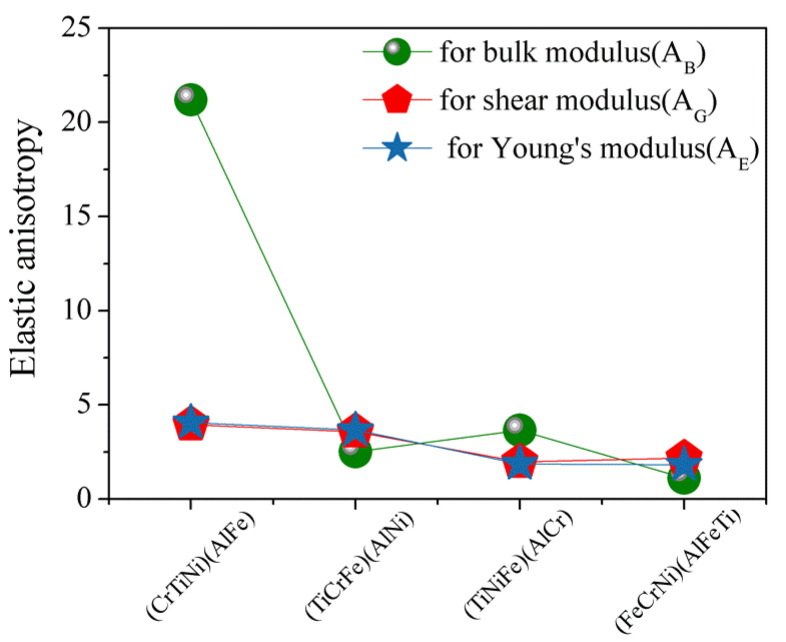
The calculated results of the percentage of anisotropy in bulk, shear, and young’s modulus for single-phase AlCrTiFeNi of different atom occupancy positions.

The elastic anisotropy of crystals plays an important role in that it results in the influence of multiaxial stress states on microcrack nucleation of materials [44]. The most widely used method for investigating elastic anisotropy is the percentage of elastic anisotropy for bulk modulus ($A_{B}$) and shear modulus ($A_{G}$) in polycrystalline materials by the following Equation [45]: $A_{B}=\frac{B_{V}-B_{R}}{B_{V}+B_{R}},\;A_{G}=\frac{G_{V}-G_{R}}{G_{V}+G_{R}}$. The value of 0 means that the crystal structure is the isotropy, while 100% suggests the maximum anisotropic of materials. The calculated results (Figure 5) for the percentage of elastic anisotropy in compression, shear, and young for (FeCrNi)(AlFeTi) atom occupancy position show that this alloy is isotropy, which indicates that AlCrTiFeNi presents an obvious elastic isotropic.

**Figure 6:**
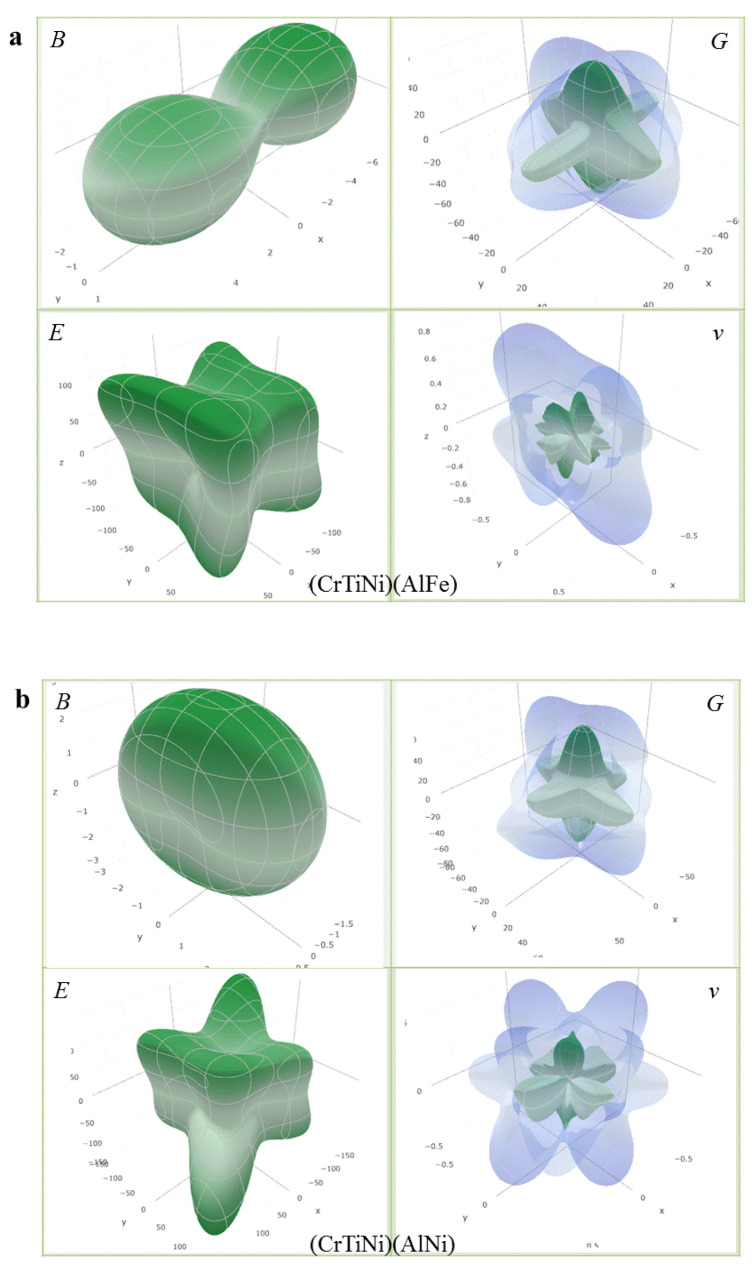

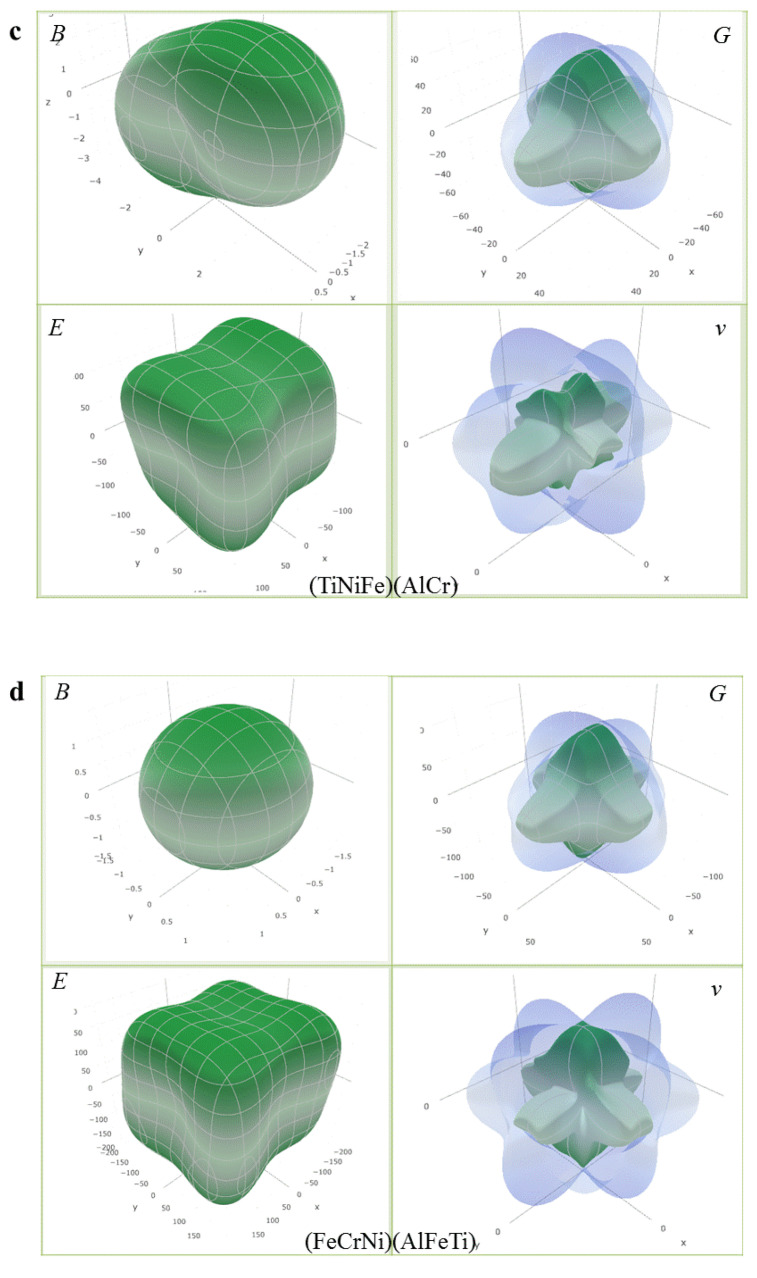
The calculated three-dimensional profile of bulk modulus (*B*), Shear modulus (*G*), elastic modulus (*E*) and poisson’s ratio (*ν*) for single-phase AlCrTiFeNi of different atom occupancy positions. (**a**) (TiCrNi)(AlFe); (**b**) (TiCrFe)(AlNi); (**c**) (TiCrFe)(AlCr); (**d**) (FeCrNi)(AlFeTi).

An alternative way to show the elastic anisotropy of a crystal is to give the direction-dependent elastic modulus (*E*) for the orthorhombic crystal structure. These expressions are defined as [46]:

$$E=1/(l_{1}{}^{4}S_{11}+l_{2}{}^{4}S_{22}+l_{3}{}^{4}S_{33}+2l_{1}{}^{2}l_{2}{}^{2}S_{12}+2l_{1}{}^{2}l_{3}{}^{2}S_{13}+2l_{2}{}^{2}l_{3}{}^{2}S_{23}+l_{1}{}^{2}l_{2}{}^{2}S_{66}+l_{1}{}^{2}l_{3}{}^{2}S_{55}+l_{2}{}^{2}l_{3}{}^{2}S_{44})$$

where ($l_{1},l_{2},l_{3}$) represent the direction cosines in the sphere coordination, ($S_{ij}$) are the elements in the compliance tensor of AlCrTiFeNi alloy, which is the inverse of the elastic tensor (C_ij_). The calculated direction-dependent elastic modulus for AlCrTiFeNi of different atom occupancy positions is shown in Figure 5, which shows that (FeCrNi)(AlFeTi) atom occupancy position is isotropic, and the (FeCrNi)(AlFeTi) atom occupancy position is serious than others. These results were obtained using open-source software ELATE calculations [47]. Figure 6 is the calculated three-dimensional profile of the bulk modulus (B), shear modulus (G), elastic modulus (E) and Poisson’s ratio (ν) of single-phase AlCrTiFeNi with different atomic positions.

### 3.4. Experimental Verification

The elastic modulus of inter-dendritic and dendritic in our experiments by the Nano-indentation test is *E*_1_ = 232.3 GPa and *E*_2_ = 236.5 GPa, respectively. These values are similar to the prediction by density functional theory calculations using Voigt-Reuss-Hill approximations (*E*_V_ = 236.55 GPa, *E*_R_ = 218.69 GPa, *E*_H_ = 227.66 GPa). Selected mechanical properties were analyzed using AlCrTiFeNi multi-principle element alloys samples. The achieved hardness was approximately 735.2 HV_0.5_ and the compressive strength 1352.2 MPa, breaking strength 1870.0 MPa, and compression ratio is 4.8%.

The HVS-1000 digital microhardness tester was used to test the microhardness of AlCrTiFeNi multi-principal alloy. The test coefficient of the hardness test is 0.5 kgf, and the loading time is 10 s. First, the grinding and polishing process is performed. To ensure the hardness test, five different positions of the alloy fragments are selected for hardness measurement. The test results are 735.26, 735.28, 735.25, 735.26, 734.95 MPa, and finally, 735.2 MPa is taken as the result of microhardness.

Use AG-X Plus 20 kN/5 kN mechanical properties testing machine to test the room temperature compression properties of AlCrTiFeNi multi-principal alloy. Cut 2–3 small cylinders of Φ 6 mm × 9 mm at different positions of each button material. A button material needs to be subjected to three compression tests, and the average value of multiple tests is taken as the test result of the room temperature compression performance. Grind the line cutting marks on the upper and lower end surfaces of the sample, and maintain the flatness of the upper and lower end surfaces. The test load is 4500 kg, and the loading speed is 0.5 mm/min. The yield strength is the yield limit when the metal material yields, that is, the stress that resists minor plastic deformation. For metal materials with no obvious yield phenomenon, the stress value that produces 0.2% residual deformation is specified as its yield limit, which is called conditional yield limit or yield strength. This article is based on the following formula:

$$\varepsilon =e^{\varepsilon_{t}}-1$$

$$\sigma =\frac{\sigma_{t}}{e^{\varepsilon_{t}}}$$

The real stress and real strain uniformly converted into engineering stress and engineering strain, where ε and σ are engineering strain and engineering stress, and $\varepsilon_{t}$ and $\sigma_{t}$ are real strain and real stress. The calculated yield strength is 1352.2 MPa, the compressive fracture strength is 1870.0 MPa, the compression rate is 12.0%, and the amount of plastic strain is 4.8%.

#### Microstructure Characterization

**Figure 7:**
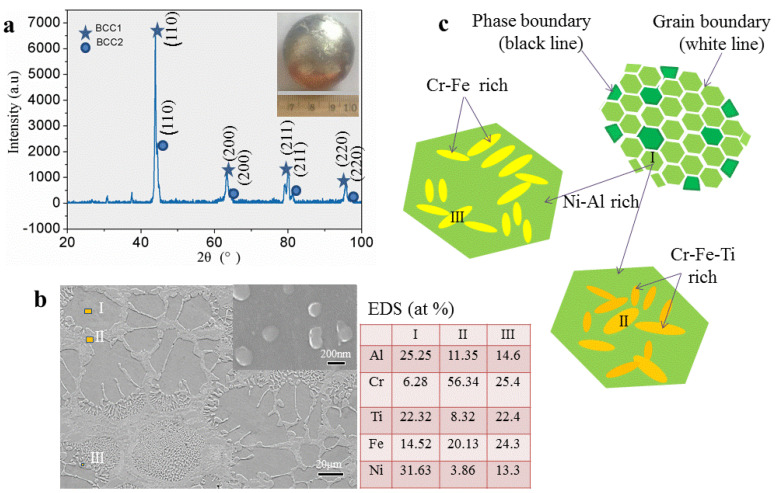
BSE images of selected AlCrTiFeNi alloy. (**a**) X-ray pattern sample; (**b**) SEM microstructure; (**c**) The schematics of the microstructure.

Based on the previous prediction of the crystal structure, we selected the AlCrTiFeNi alloy whose atomic composition is equimolar ratio for detailed experimental research. The X-ray patterns of the corresponding as-cast sample (Figure 7a) indicates that the bcc1 and bcc2 phases have microstructures with unit cell parameters of a = 2.9533 Å and a = 2.9250 Å, respectively. This crystal structure parameter is like the calculated lattice constants (a = 2.866) of (FeCrNi)(AlFeTi) atom occupancy positions, which indicates that the calculation is exceptionally reliable.

The SEM image of the specimens in Figure 7b shows that it is similar to the dendrite stem microstructure of many other alloys, the patterns consist with dendritic (DR) structure (the position I) and inter-dendritic structure (ID) (positions II and III), and areas of Nano-phases between the dendritic (with size about 200 nm). Energy dispersive spectroscopy (EDS) analysis shows that Cr tends to segregate within the DR, while Ni and Al tend to assemble between the inter-dendritic. The compositional distribution of the eutectic structure is closer to the average of DR and ID structures components. As Figure 7c shows the schematics of the microstructure, this morphology consists of Ni-Al rich (the position I) bcc solid solution, Cr-Fe rich (position II), and Cr-Fe-Ti rich (position III) bcc solid solution and it exists component segregation.

**Figure 8:**
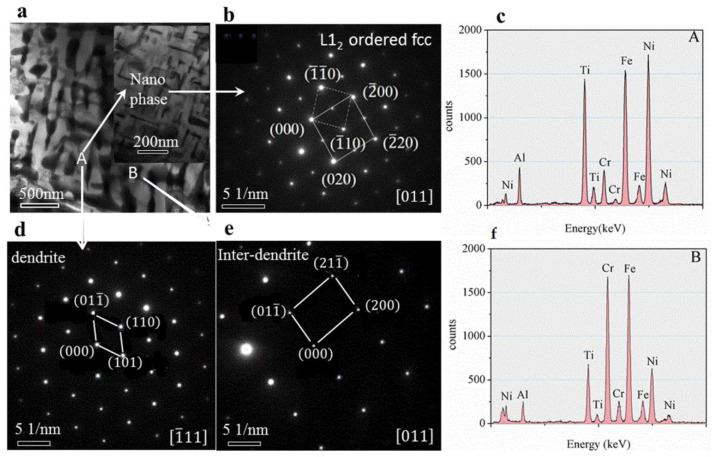
(**a**–**c**) TEM microstructures and Nano phase, (**d**,**e**) The selected area electron diffraction (SAED) pattern from dendrite and Inter-dendrite, respectively. (**f**) EDS energy spectrum.

The TEM bright-field image of the as-cast microstructure (Figure 8a) shows that dendritic (Figure 8d) and inter-dendritic (Figure 8e) structures (Selected Area Electron Diffraction pattern) with bcc1 and bcc2 phases, and the tiny Nano-phases distributed within the dendrites (Figure 8b) with size about 200 nm, the structure was L_12_ type ordered fcc phase. The calibration results showed that the lattice constant of the ordered Nanostructure is a = 5.8926 Å. Analysing the characteristics of the geometric relationship between the two sets of diffraction patterns, it can be concluded that the Nano-precipitate and crystal structure of inter-dendritic matrix satisfies the following phase relationship: (α represent the matrix of dendritic, α′ represent the matrix of nano-precipitates): ${\left\{220\right\}}_{\alpha }//{\left\{220\right\}}_{\alpha^{\prime }}\;{\left\{200\right\}}_{\alpha }//{\left\{200\right\}}_{\alpha^{\prime }}$, ${\langle 100\rangle }_{\alpha }//{\langle 100\rangle }_{\alpha^{\prime }}$. The concentrations as shown in Figure 8c (obtained from TEM-EDS) indicate a small segregation of Fe, Ni, and Ti elements in the dendritic of matrix and Fe and Cr elements in the inter-dendritic. These small concentration variations indicate the presence of two types of phases, consistent with the results of XRD and SEM analyses.

## 4. Conclusions

In summary, we used a material genetic engineering approach to study crystal structure, atom occupancy position, deformation behavior, and micro-mechanism for the AlCrTiFeNi multi-principle element alloy. Using a theoretical prediction, the atomic-size difference δ = 7.212, mixing enthalpy $\Delta H_{mix}$ = −0.24 KJ/mol, and VEC = 6.20 (bcc structure) criterion simultaneously, we can choose a bcc-structured solid solution for designing AlCrTiFeNi multi-principle element alloys easily and effectively. The total energy $E_{\mathrm{tot}}$, formation enthalpy $H_{f}$, cohesive energy $E_{c}$ and secondary solid solution formation enthalpy $H_{c}^{c}$ in the single-phase AlCrTiFeNi multi-principle element alloy showed that the (FeCrNi)(AlFeTi) is probably one of the most stabilized atom occupancy positions of bcc phase AlCrTiFeNi. For the equiatomic AlCrTiFeNi multi-principle element alloy, the experiment verified that the structure consisted of inter-dendritic and dendritic structures with bcc1 and bcc2 phases, and as the tiny nanophases were distributed within the dendrites with a size of about 200 nm, the structure was L12 type ordered fcc phase. Further analysis of the characteristics of the microstructure included the decomposition of solid solution. The proposed approach reproduced the fundamental component atoms and structural properties (lattice parameter, structural stability, and elastic modulus) of the AlCrTiFeNi multi-principle element alloy in good agreement with experimental information. This approach can be further utilized for a more elaborate alloy and the process design of a new multi-principle element alloy with targeted properties.
